# Immigration and C-sections incidence: Maternal care and perinatal outcomes in the context of the pandemic in Chile

**DOI:** 10.3389/fgwh.2023.1267156

**Published:** 2023-11-22

**Authors:** María Begoña Carroza Escobar, Nicole Silva, Jovita Ortíz-Contreras, Rodrigo Villegas, Sergio L. Vargas, Claudio Núñez, Luis Felipe Vergara Maldonado, Loreto Paola Villanueva

**Affiliations:** ^1^ Programa de Doctorado en Salud Pública, Escuela de Salud Pública, Facultad de Medicina, Universidad de Chile, Santiago, Chile; ^2^ Departamento de Promoción de la Salud de la Mujer y el Recién Nacido, Facultad de Medicina, Universidad de Chile, Santiago, Chile; ^3^ Servicio de Ginecología y Obstetricia, Complejo Hospitalario San José, Servicio de Salud Metropolitano Norte, Santiago, Chile; ^4^ Programa de Bioestadística, Escuela de Salud Pública, Facultad de Medicina, Universidad de Chile, Santiago, Chile; ^5^ Programa de Microbiología, Instituto de Ciencias Biomédicas, Facultad de Medicina, Universidad de Chile, Santiago, Chile; ^6^ Programa de MBA con Especialización en Salud, Instituto de Salud Pública, Universidad Andrés Bello, Santiago, Chile

**Keywords:** COVID, immigrant women, cesarean sections, maternal care, pregnancy, perinatal outcomes

## Abstract

**Introduction:**

Immigration has increased significantly in Chile. Despite that all pregnant women, regardless of nationality and immigration status, have the right to access to all healthcare services during pregnancy, childbirth, and postpartum, inequities in health care outcomes and health provision have been reported. During COVID-19 pandemic, these inequities are completely unknown.

**Objective:**

The aim of this study was to compare the incidence of c-sections according to mother's migration status, as well as other maternal care and perinatal outcomes in women giving birth at San José Hospital in Santiago, Chile, during the COVID-19 pandemic.

**Methods:**

A retrospective cohort study was designed including 10,166 registered single births at the San José Hospital between March 2020 and August 2021. To compare between groups, statistical tests such as Chi-square and Fisher's exact were used. Log Binomial regression models were performed adjusted for potential confounding variables. To estimate the strength of association the relative risk was used.

**Results:**

Immigrant mothers account for 48.1% of the registered births. Compared to non-immigrant women, immigrants exhibit a higher proportion of c-section, specifically, emergency c-section (28.64% vs. 21.10%; *p*-value < 0.001) but a lower proportion of and having a preterm birth (8.24% vs. 13.45%; *p* < 0.05), receiving personalized childbirth care (13.02% vs. 14.60%; *p*-value < 0.05), companion during labor and childbirth (77.1% vs. 86.95%; *p*-value < 0.001), And postpartum attachment to newborn (73% vs. 79.50%; *p*-value < 0.001). The proportion of COVID exposure was not significant between groups, not the severity also. Haitians had a highest risk of undergoing emergency c-section (aRR = 1.61) and Venezuelans had a highest risk of elective c-section (aRR = 2.18) compared to non-immigrants.

**Conclusion:**

This study reports high rates of c-sections in the entire population, but in immigrant populations it is even higher. Additionally, it found gaps in maternal care and perinatal outcomes between immigrants and non-immigrants. More studies are needed to elucidate the possible causes of these differences and establish new regulations to protect the reproductive rights of the immigrant population.

## 1. Introduction

Immigration has increased significantly in Chile. In 20 years, it went from being 0.8% in 1992, to 7.5% in 2021 (1), however, due to their demographic characteristics, immigrants' mothers account for the 16.4% of live births, with the top five maternal nationalities being Haiti (21.6%), Venezuela (17.1%), Peru (12.5%), Bolivia (7.9%), and Colombia (6.8%) (2). Some international studies have reported higher C-section rates among the immigrant population compared to the local population (3, 4). While the causes for these higher rates remain unknown, certain risk factors have been identified, such as language or communication barriers, inadequate prenatal care even after a shorter stay in the host country, socioeconomic status, and lack of health insurance (5, 6).

Chile is one of the few countries in Latin America and the Caribbean that has reached the advanced stage of the obstetric transition (7). One of the major challenges at this stage is the excessive obstetric interventionism and medicalization of childbirth, as a consequence, Chile has one of the highest rates of c-sections in the region, reaching 43.1% of total births in the public system in 2020 (8). Moreover, an increasing rate of obesity in women of childbearing age (9, 10), delayed pregnancy, and barriers to access to healthcare for the immigrant population (11–13), are urgent priorities to be addressed.

COVID-19 has been associated with an increase in adverse maternal and perinatal health conditions, such as preterm birth and maternal hospitalization (14, 15). According to Haye et al., during the pandemic, there was an observed increase in C-section rates (16). However, it remains unclear whether this increase is due to the perinatal consequences of COVID-19 or changes in medical decision-making regarding C-section practice, or the impact of other factors such as immigration (17).

In Chile, pregnant women, regardless of nationality and immigration status, have access to all healthcare services during their pregnancy, childbirth, and up to 12 months postpartum within the public system (18). Despite immigrants having the same rights as the non-immigrant population, the inequities in health care and health outcomes are unknown.

At the beginning of the pandemic period, hospitals modified protocols to reduce the spread of the virus, so the maternal care provision changed, visitors were prohibited, companionship during labor and childbirth was reduced and the attachment to the baby was restricted in mothers with COVID-19. Due to the concern of hospital collapse because of the excess of COVID-19 cases and high levels of stress or depression observed in mothers giving birth (19), providing a positive birth experience was a challenge.

The aim of this study was to compare the incidence of C-sections, obstetric and neonatal outcomes according to the mother's migration status when giving birth at San José Hospital in Santiago, Chile, during the COVID-19 pandemic.

## 2. Methods

### 2.1. Study design and population

**Figure 1:**
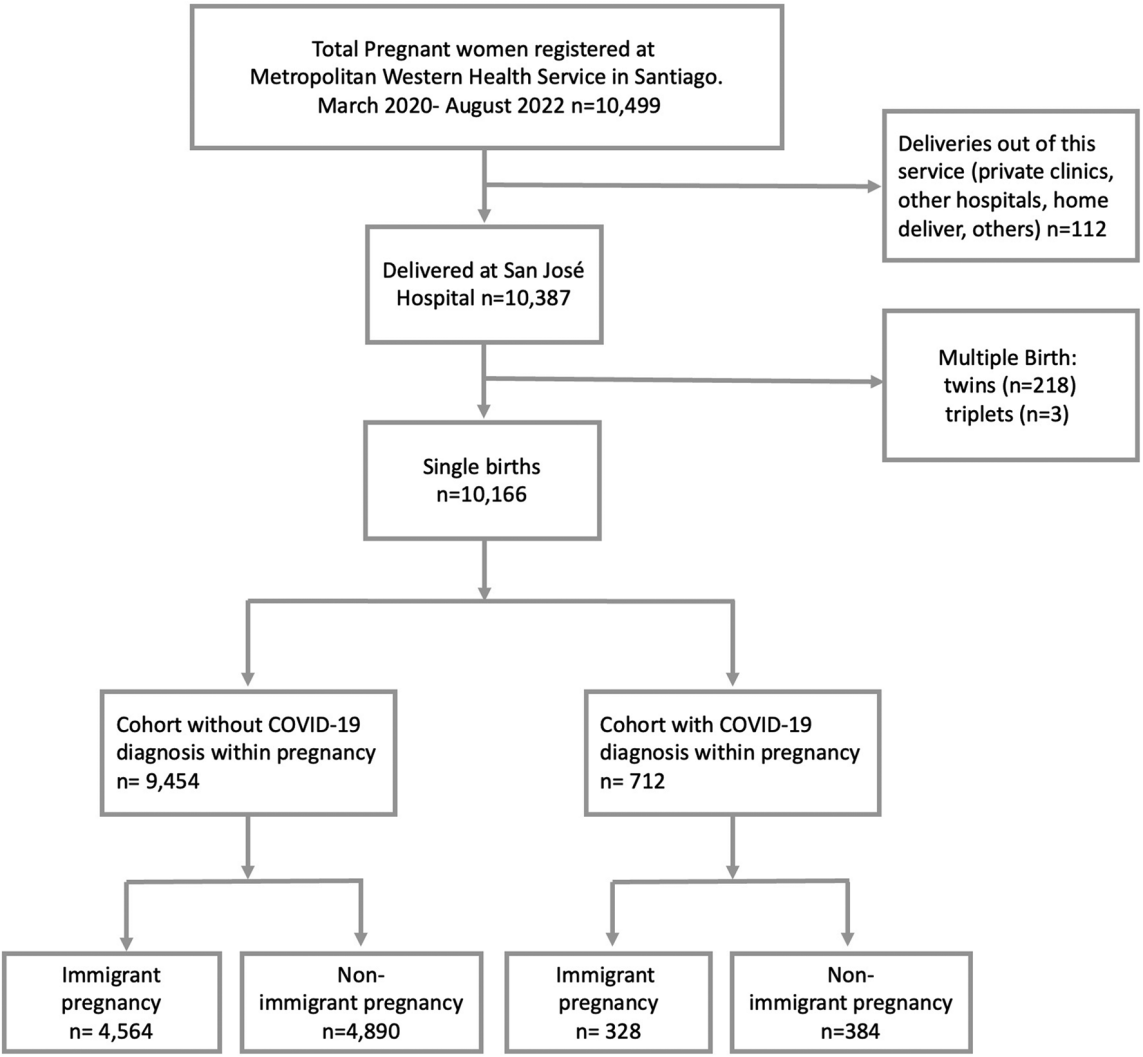
Universe and sample included in this cohort study. Santiago, Chile.

A retrospective cohort study was designed including 10,166 registered single births at the *San José Hospital* between March 2020 (pandemic's starting date in Chile) and August 2021 (before the vaccination of pregnant women began) (Figure 1). The San José Hospital is a high complexity public's health provider, serving a population of approximately 1.2 million. Nearly 50% of pregnant women are immigrants according to previous reports, with the most common nationalities being Haiti, Venezuela, and Peru. The data was obtained from the systematized clinical records. The diagnosis of COVID-19 was made using Polymerase Chain Reaction (PCR). At the beginning of the pandemic, the PCR test was performed as screening on patients with clinical symptoms. Subsequently, from May 2020 the PCR was taken preventively to all hospitalized patients admitted to the hospital. That means that asymptomatic patients could be undiagnosed, in consequence, a slight underestimation of the parameter within the first two months is expected.

### 2.2. Instrument and variables

The data was gathered from the hospital's clinical record and the women's health agenda, which includes historical records of pregnancy check-ups.

#### 2.2.1. Exposure variables

The data was analyzed in terms of non-immigrant and immigrant status (non-immigrant = 0, immigrant = 1). Then, specific analyses were conducted considering the most prevalent nationalities, pair comparison such as Haitians with non-immigrants, Venezuelan with non-immigrants, and finally, immigrants from other nationalities were included in comparison to non-immigrants.

#### 2.2.2. Outcome variables

The main outcome variable of this study was the type of delivery, categorized as vaginal birth, forceps-assisted vaginal birth, elective c-section, and emergency c-section. For a separate analysis of elective c-section and emergency c-section categories, vaginal birth (including forceps-assisted births) was used as the reference.

Maternal care variables are personalized childbirth care, labor and childbirth companion, postpartum attachment to newborns (defined as the immediate connection that occurs once the baby is born, involving skin-to-skin contact with the mother and the promotion of the first breastfeeding within the first 30 min and a maximum of one hour) (20). Finally, neonatal variables include newborn sex (female, male, undefined), Apgar score, gestational weeks at birth, and newborn weight and height.

#### 2.2.3. Covariables

**Figure 2:**
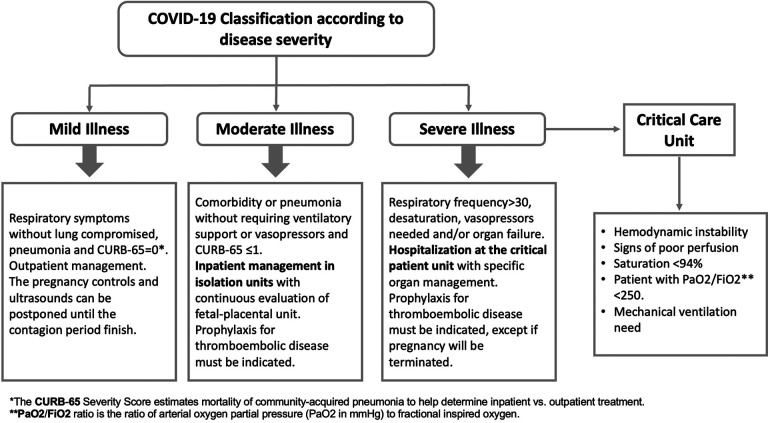
Case management protocol of SARS-CoV-2 virus (COVID-19) during pregnancy, puerperium, and mother-baby dyad care at the San José hospital, in Santiago of Chile.

Clinical variables related to COVID-19 include severity of exposure illness (non-COVID, mild illness, moderate illness, and severe illness) (Figure 2), critical patient unit (CPT), pneumonia, mechanical ventilation, prone position, pulmonary embolism (TEP), and hospitalization based on disease severity. Other maternal covariables are maternal obesity and labor induction.

Sociodemographic variables include age (in years) and type of health insurance (FONASA is the State-run health service founded by public taxes and the 7% of the income deduction; private insurance is funded using the contributions of their members).

### 2.3. Statistical analysis

Categorical variables were presented as frequency and percentage, while quantitative variables were categorized. To compare between groups, statistical tests such as Chi-square and Fisher's exact test (with less than 5 observations) were used, considering a *p*-value <0.05 as significant. Log Binomial multivariable regression models were used to calculate risk ratios (RRs) crude and adjusted for potential confounding variables to estimate the strength of association between nationality variable and types of c-section (elective and emergency). The data were analyzed using Stata software version 18.

## 3. Results

10,166 births were registered at the San José Hospital within the study period. The immigrant population was 48.12% (*n* = 4,892), the most prevalent countries of origin were Haiti 36.41% (*n* = 1,781), Peru 24.9% (*n* = 1,218), and Venezuela 19.91% (*n* = 974).

Immigrants have a lower percentage of women under 19 years-old compared to non-immigrant group (4.03% vs. 8.76% respectively; *p* < 0.05) and a higher proportion of women aged 35 years-old and more, although this difference was not statistically significant.

A significant difference was found in the type of health insurance between the two groups. Immigrants register a higher percentage of enrollment to FONASA A compared to non-immigrants (80.09% vs. 55.46%, respectively), but a lower proportion of enrollment to FONASA B and C compared to non-immigrant women. Non-significant differences were found in private health insurance (ISAPRE) between the groups.

Regarding parity, a higher percentage of immigrant women were primiparous compared to non-immigrants (38.88% vs. 30.24% respectively; *p* < 0.05). A significant difference was found in body mass index (BMI) between the two groups. Immigrants has a lower percentage of obesity (BMI 30 or more), compared to non-immigrant women (53.85% vs. 70.05%, respectively; *p*-value <0.001). No significant differences were found in the prevalence of COVID-19 between both groups (Table 1).

**Table 1:** Sample characterization. Cohort of 10,166 single births at the hospital San José in Santiago of Chile between March 2020 to August 2022.

	Non-immigrant pregnancy (*n* = 5,274)	Immigrant pregnancy (*n* = 4,892)	Total	*p*-value*
	n (%)	*n* (%)	*n* (%)	
Age				
<19 years-old	462 (8.76)	197 (4.03)	659 (6.49)	<0.000*
20 to 34 years-old	3,918 (74.30)	3,756 (76.86)	7,674 (75.53)	
35 and more	893 (16.94)	934 (19.11)	1,827 (17.98)	
COVID				
Non exposed to COVID	4,890 (97.72)	4,564 (93.30)	9,454 (93)	<0.255
Exposed to COVID	384 (7.28)	328 (6.70)	712 (7.0)	
Health Insurance**				
FONASA A	2,924 (55.46)	3,918 (80.09)	6,842 (67.32)	<0.000*
FONASA B	999 (18.95)	347 (7.09)	1,346 (13.24)	
FONASA C	568 (10.77)	347 (7.09)	915 (9.00)	
FONASA D	607 (11.51)	203 (4.15)	810 (7.97)	
Private (ISAPRE)	77 (1.46)	77 (1.57)	154 (1.52)	
Parity				
Primipara	1,595 (30.24)	1,902 (38.88)	3,497 (34.40)	<0.000*
Multipara	3,679 (69.76)	2,990 (61.12)	2,990 (61.12)	
Obesity preganancy				
BMI < 30	436 (29.95)	623 (46.15)	1,059 (37,74)	<0.000*
BMI ≥ 30	1,020 (70.05)	727 (53.85)	1,747 (62.26)	

* Statistically significant *p*-value <0.05 *χ*^2^ test.

** FONASA is the public system and is funded by taxes, providing free or subsidized care for those who cannot afford private health insurance. Classification according to FONASA in 2020. https://www.fonasa.cl/sites/fonasa/noticia/nuevos_tramos_Fonasa_2020 FONASA A (lack income or a formal job); FONASA B (income equal to or less than the minimum wage $319,000 Chilean peso in March 2020); FONASA C (Monthly income greater than $319,000.—and less than or equal to $465,740 Chilean pesos); FONASA D (People who receive a monthly taxable income greater than $465,740 Chilean peso).

Immigrant women had a higher proportion of c-sections compared to non-immigrant women, specifically emergency c-sections (28.64% vs. 21.10%; *p*-value <0.001). In contrast, vaginal births were more frequent in non-immigrant women (69.6%).

Regarding maternal care provision, immigrant women registered a significantly smaller percentage of receiving personalized care or having a companion compared to non-immigrant women, and had less postpartum attachment with their babies, compared to immigrant women. Furthermore, immigrants have a lower proportion of preterm births compared with non-immigrants (*p*-value <0.001) (Table 2).

**Table 2:** Comparison of obstetric and perinatal outcomes between non-immigrant and immigrant pregnant women in the context of a COVID-19 pandemic. Hospital San José, Santiago of Chile. March 2020 to August 2022.

Birth outcomes	Non-immigrant pregnancy (*n* = 5,274)	Immigrant pregnancy (*n* = 4,892)	Total	*p*-value*
	*n* (%)	*n* (%)		
Conduct of labor				
No	2,458 (46.61)	2,272 (46.44)	4,730 (46.53)	<0.869
Yes	2,816 (53.39)	2,620 (53.56)	5,436 (53.47)	
Induction of labor				
No	4,367 (82.80)	4,053 (82.85)	8,420 (82.83)	<0.950
Yes	907 (17.20)	839 (17.15)	1,746 (17.17)	
Monitoring of labor				
No	803 (15.23)	727 (14.86)	1,530 (15.05)	<0.607
Yes	4,471 (84.77)	4,165 (85.14)	8,636 (84.95)	
Personalized childbirth care				
No	4,504 (85.40)	4,255 (86.98)	8,759 (86.16)	<0.021*
Yes	770 (14.60)	637 (13.02)	1,407 (13.84)	
Labor and childbirth companion				
No	688 (13.05)	1,120 (22.89)	1,808 (17.78)	<0.000*
Yes	4,586 (86.95)	3,772 (77.11)	8,358 (82.22)	
Birth type of delivery				
Vaginal birth	3,671 (69.61)	3,005 (61.43)	6,676 (65.67)	<0.000*
Forceps-assited birth	64 (1.21)	66 (1.35)	130 (1.28)	
Elective c-section	426 (8.08)	420 (8.59)	846 (8.32)	
Emergency c-section	1,113 (21.10)	1,401 (28.64)	2,524 (24.73)	
Fetal position at birth				
Cephalic	5,054 (95.90)	4,738 (96.91)	9,792 (96.32)	<0.024*
Podalic	193 (3.67)	136 (2.79)	329 (3.24)	
Transverse	23 (0.44)	15 (0.31)	38 (0.37)	
Postpartum attachment to newborns				
No	1,081 (20.50)	1,321 (27.00)	2,402 (23.63)	<0.000*
Yes	4,193 (79.50)	3,571 (73.00)	7,764 (76.37)	
Newborn condition				
Live birth	5,240 (99.36)	4,846 (99.06)	10,086 (99.21)	<0.092
Stillbirth	34 (0.64)	46 (0.94)	80 (0.79)	
Maternal mortality				
No	5,270 (99.92)	4,889 (99.94)	10,159 (99.93)	<0.541
Yes	4 (0.08)	3 (0.06)	7 (0.07)	Fisher's exact
Classification by gestational weeks				
Term (≥37 Weeks)	4,570 (86.65)	4,489 (91.76)	9,059 (89.11)	<0.000*
Moderate (32–36 weeks)	582 (11.04)	304 (6.21)	886 (8.72)	
Very Preterm (28–31 Weeks)	79 (1.50)	43 (0.88)	122 (1.20)	
Extremely (<28 weeks)	43 (0.82)	56 (1.14)	99 (0.97)	
Classification by birthweight				
Normal	4,760 (90.25)	4,485 (91.68)	9,245 (90.94)	<0.001*
Low birthweght	420 (7.96)	311 (6.36)	731 (7.19)	
Very low birtweight	53 (1.00)	37 (0.76)	90 (0.89)	
Extremely low birthweight	41 (0.78)	59 (1.21)	100 (0.98)	
APGAR at 1 min				
Normal (>7)	5,037 (95.51)	4,604 (94.11)	9,641 (94.84)	<0.006*
Moderate depression (4–6)	120 (2.28)	152 (3.11)	272 (2.68)	
Severe depression (<4)	117 (2.22)	136 (2.78)	253 (2.49)	
Apgar at 5 min				
Normal (>7)	5,188 (98.37)	4,793 (97.98)	9,981 (98.18)	<0.296
Moderate depression (4–6)	30 (0.57)	38 (0.78)	68 (0.67)	
Severe depression (<4)	56 (1.06)	61 (1.25)	117 (1.15)	

* Statistically significant *p*-value < 0.05 *χ*^2^ test.

Regarding the COVID-19 no differences were found in the severity of illness, need for admission to the Intensive Care Unit (ICU), the presence of pneumonia, the use of mechanical ventilation, prognosis complications, pulmonary embolism (PE), between the two groups (Table 3).

**Table 3:** Comparison of COVID clinical severity between non-immigrant and immigrant pregnant women. Hospital San José, Santiago of Chile. March 2020 to August 2022.

Outcomes COVID	Non-immigrant pregnancy (*n* = 5,274)	Immigrant pregnancy (*n* = 4,892)	Total	*p*-value*
	*n* (%)	*n* (%)		
Severity of exposure illness				
Non COVID	4,890 (92.72)	4,564 (93.30)	9,454 (93.00)	<0.457
Mild illness	336 (6.37)	295 (6.03)	631 (6.21)	
Moderate illness	12 (0.23)	10 (0.20)	22 (0.22)	
Severe illness	36 (0.68)	23 (0.47)	59 (0.58)	
Critical Patient Unit (CPT)				
No	5,238 (99.32)	4,869 (99.53)	10,107 (99.42)	<0.159
Yes	36 (0.68)	23 (0.47)	59 (0.58)	
Pneumonia				
No	5,232 (99.20)	4,863 (99.41)	10,095 (99.30)	<0.218
Yes	42 (0.80)	29 (0.59)	71 (0.70)	
Mechanical ventilation				
No	5,255 (99.64)	4,882 (99.80)	10,137 (99.71)	<0.141
Yes	19 (0.36)	10 (0.20)	29 (0.29)	
Prone				
No	5,269 (99.91)	4,891 (99.98)	10,160 (99.94)	<0.221
Yes	5 (0.09)	1 (0.02)	6 (0.06)	Fisher's exact
Pulmonary Embolism				
No	5,264 (99.81)	4,887 (99.90)	10,151 (99.85)	<0.307
Yes	10 (0.19)	5 (0.10)	15 (0.15)	Fisher's exact
Hospitalization				
No	5,154 (97.74)	4,764 (97.40)	9,918 (97.58)	<0.623
Yes	119 (2.26)	127 (2.60)	246 (2.42)	

* Statistically significant *p*-value < 0.05 *χ*^2^ test.

**Figure 3:**
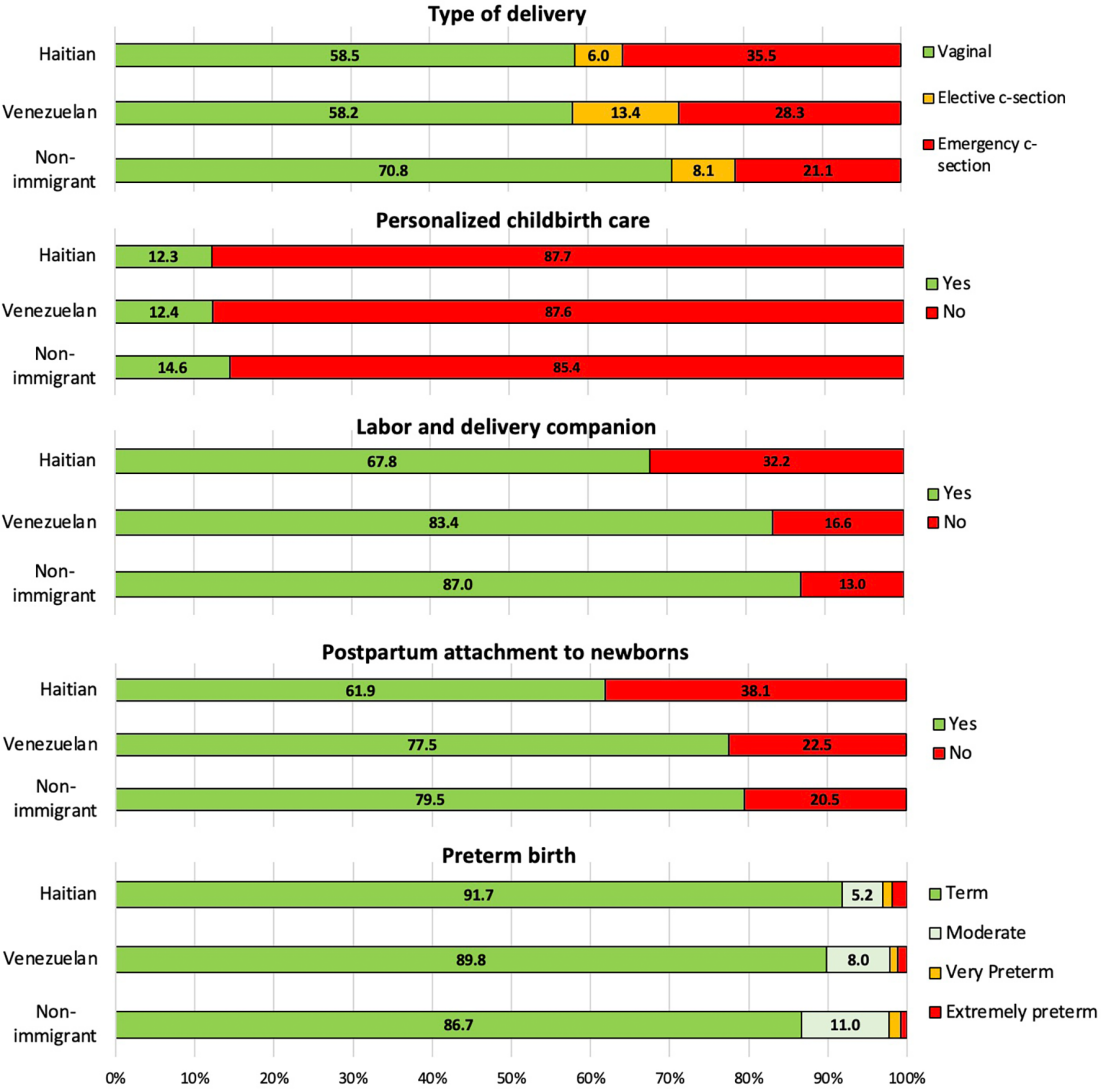
Proportion of maternal care indicators and perinatal outcomes by country of origin.

As shown in Figure 3, Haitians had the highest proportion of emergency c-section and preterm birth, but the lowest proportion of labor and delivery companion, postpartum attachment to newborn. On the other hand Venezuelans had the highest proportion of elective c-sections and less proportion of maternal care provision variables compared with non-immigrants (Figure 3).

In the pair comparison analysis shown in Table 4, Haitians had a higher risk of undergoing emergency c-section compared to non-immigrants (aRR = 1.61; 95% CI: 1.48–1.75). Venezuelans, had a higher risk of both planned/elective c-section and emergency c-section compared to non-immigrants (aRR = 2.18; 95% CI: 1.81–2.63 and aRR = 1.50; 95% CI: 1.33–1.68, respectively). Women from other nationalities, exhibited a slightly higher risk of emergency c-section was reported but after adjusting by age and health insurance, the risk of emergency c-section disappeared (aRR = 1.09; 95% CI: 0.99–1.19). Both Haitians and Venezuelans had a significant higher risk of not having maternal care provision, however, Haitians had a higher risk of not receiving personalized childbirth care (aRR = 1.03; 95% CI: 1.00–1.05), not having a companion during labor and childbirth (aRR = 2.48; 95% CI: 2.24–2.75), not having postpartum attachment to newborns (aRR = 1.88; 95% CI: 1.73–2.05). Also Haitians has the highest risk of extreme preterm birth (<28 weeks, aRR = 2.09; 95% CI: 1.30–3.36) compared to non-immigrants. (Table 4).

**Table 4:** Risk of c-section and other obstetric and perinatal outcomes in Haitian, Venezuelan, and other nationalities, compared to non-immigrant women. Log Binomial multivariable regression models.

Birth outcomes	Non-immigrant pregnancy (*n* = 5,274)	Haitian pregnancy (*n* = 1,781) (A)			Venezuelan pregnancy *n* = 974) (B)			Other Immigrant pregnancy (*n* = 2,137) (C)		
	*n* (%)	*n* (%)	RR (IC95%)	RRa (IC95%)	*n* (%)	RR (IC95%)	RRa (IC95%)	*n* (%)	RR (IC95%)	RRa (IC95%)
Birth type of delivery										
Vaginal birth	3,735 (70.82)	1,042 (58.51)	Ref	Ref	597 (58.21)	Ref	Ref	1,462 (68.41)	Ref	Ref
Elective cesarean section	426 (8.08)	107 (6.01)	0.93 (0.74–1.11)	0.96 (0.78–1.18)	131 (13.45)	1.83 (1.53–2.19)	2.18 (1.81–2.63)	182 (8.52)	1.08 (0.92–1.27)	1.11 (0.94–1.31)
Emergency cesarean section	1,113 (21.10)	632 (35.49)	1.64 (1.52–1.78)	1.61 (1.48–1.75)	276 (28.34)	1.43 (1.28–1.59)	1.50 (1.33–1.68)	493 (23.07)	1.09 (1.00–1.20)	1.09 (0.99–1.19)
Personalized childbirth care										
No	4,504 (85.40)	1,562 (87.70)	1.02 (1.01–1.05)	1.03 (1.00–1.05)	853 (87.58)	1.03 (0.99–1.52)	1.03 (1.05–1.06)	1,840 (86.10)	1.01 (0.99–1.03)	1.01 (0.99–1.03)
Yes	770 (14.60)	219 (12.30)	Ref	Ref	121 (12.42)	Ref	Ref	297 (13.90)	Ref	Ref
Labor and childbirth companion										
No	688 (13.05)	574 (32.23)	2.47 (2.24–2.72)	2.48 (2.24–2.75)	162 (16.63)	1.27 (1.09–1.49)	1.32 (1.11–1.56)	384 (17.97)	1.38 (1.23–1.54)	1.35 (1.20–1.52)
Yes	4,586 (86.95)	1,207 (67.77)	Ref	Ref	812 (83.37)	Ref	Ref	1,753 (82.03)	Ref	Ref
Postpartum attachment to newborns										
No	1,081 (20.50)	678 (38.07)	1.86 (1.72–2.01)	1.88 (1.73–2.05)	219 (22.48)	1.09 (0.96–1.25)	1.15 (1.00–1.31)	424 (19.84)	0.97 (0.88–1.07)	0.98 (0.88–1.08)
Yes	4,193 (79.50)	1,103 (61.93)	Ref	Ref	755 (77.52)	Ref	Ref	1,713 (80.16)	Ref	Ref
Classification by gestational weeks										
Term (≥37 Weeks)	4,570 (86.65)	1,634 (91.75)	Ref	Ref	875 (89.84)	Ref	Ref	1,980 (92.65)	Ref	Ref
Moderate (32–36 weeks)	582 (11.04)	92 (5.17)	0.47 (0.38–0.58)	0.51 (0.41–0.64)	78 (8.01)	0.72 (0.58–0.91)	0.85 (0.68–1.08)	134 (6.27)	0.56 (0.47–0.67)	0.59 (0.49–0.71)
Very Preterm (28–31 Weeks)	79 (1.50)	22 (1.24)	0.78 (0.49–1.25)	0.85 (0.52–1.38)	9 (0.92)	0.60 (0.30–1.19)	0.72 (0.36–1.47)	12 (0.56)	0.35 (0.19–0.65)	0.34 (0.21–0.69)
Extremely (<28 weeks)	43 (0.82)	33 (1.85)	2.12 (1.35–3.33)	2.09 (1.30–3.36)	12 (1.23)	1.45 (0.77–2.74)	1.47 (0.75–2.88)	11 (0.51)	0.59 (0.31–1.15)	0.60 (0.30–1.17)
Classification by birthweight										
Normal	4,760 (90.25)	1,589 (89.22)	Ref	Ref	876 (89.94)	Ref	Ref	2,020 (94.53)	Ref	Ref
Low birthweght	420 (7.96)	136 (7.64)	0.97 (0.81–1.17)	1.03 (0.85–1.25)	77 (7.91)	0.99 (0.79–1.26)	1.13 (0.89–1.44)	98 (4.59)	0.57 (0.46–0.71)	0.59 (0.48–0.74)
Very low birtweight	53 (1.00)	24 (1.35)	1.35 (0.84–2.18)	1.63 (0.98–2.69)	7 (0.72)	0.72 (0.33–1.58)	1.18 (0.52–2.71)	6 (0.28)	0.27 (0.12–0.62)	0.32 (0.14–0.76)
Extremely low birthweight	41 (0.78)	32 (1.80)	2.31 (1.46–3.66)	2.35 (1.45–3.83)	14 (1.44)	1.84 (1.01–3.36)	2.06 (1.08–3.92)	13 (0.61)	0.75 (0.40–1.39)	0.78 (0.41–1.48)
APGAR at 1 min										
Normal (>7)	5,037 (95.51)	1,609 (90.34)	Ref	Ref	927 (95.17)	Ref	Ref	2,068 (96.77)	Ref	Ref
Moderate depression (4–6)	120 (2.28)	93 (5.22)	2.35 (1.80–3.06)	2.43 (1.83–3.22)	26 (2.67)	1.17 (0.77–1.78)	1.25 (0.81–1.94)	33 (1.54)	0.68 (0.46–0.99)	0.68 (0.46–0.99)
Severe depression (<4)	117 (2.22)	79 (4.44)	2.06 (1.56–2.73)	1.96 (1.45–2.63)	21 (2.16)	0.98 (0.62–1.54)	1.05 (0.65–1.69)	36 (1.68)	0.75 (0.52–1.09)	0.75 (0.51–1.09)

RR, Crude relative risk; RRa, adjusted relative risk by age and health insurance.

A = Haitians compared to non-immigrants (Haitians = 1, non-immigrants = 0).

B = Venezuelan compared to non-immigrants (Venezuelan = 1, non-immigrants = 0).

C = Other nationalities (except Haitians and Venezuelans), compared to non-immigrantrs (other nationalities = 1, non-immigrants = 0).

## 4. Discussion

The present study reported significant differences in perinatal outcomes between immigrant and non-immigrant women. The main finding of this study is related to high rates of c-section in the entire population, that in immigrants it exceeds 40% of the total births, which is one of the highest rates reported in the world. Haitian women had the highest risk of emergency c-section and Venezuelan women the highest rate of elective c-section. Additionally, it found a higher risk of not having a companion during labor and not postpartum attachment with the newborn among mothers from Haiti.

Regarding biosociodemographic data, the results demonstrate that approximately half of the pregnant women attended at the San José Hospital were immigrants, most prevalent countries of origin were Haiti, Peru, and Venezuela. Significant differences were observed in various demographic and health variables between immigrant and non-immigrant women. These findings are consistent with previous studies that have examined the composition of the immigrant population in Chile (11–13). A study conducted by Veliz et al. supports the presence of a significant proportion of immigrants from the same countries mentioned. This validates and enhances the representativeness of the current study's sample (21).

We found that immigrant women received less personalized attention during labor and had a lower presence of companion at birth compared to non-immigrant women. Additionally, significant differences were observed in postpartum attachment with newborns, prematurity and birth weight. These findings are consistent with previous research that has addressed the unequal impact of the pandemic on the health of migrants and the exercising of their reproductive rights. International studies have reported challenges in establishing the mother-newborn emotional connection in immigrant women, which may be related to factors such as social support, communication barriers, and cultural adaptation. A publication from Fabi and Ludmir (17), suggests that immigrant women, in particular, have experienced difficulties asserting their reproductive rights due to the pandemic. Although the pandemic could have accentuated the differences between the immigrant and non-immigrant population, more studies are required to determine the effect of migration as a social determinant of inequities in childbirth care, independent of the health crisis.

Significant differences were found in the c-section rate. Although other studies have shown higher rates of c-section in immigrant women in different countries (10, 22, 23), Chile has been recognized for its high rates of c-section. Nevertheless, the disparity in cesarean section rates between immigrant and non-immigrant populations was not known. Although it is known that there are determining factors such as access to prenatal care, communication barriers and other social determinants of health, it is urgent to design more studies to elucidate the possible causes and establish new regulations that protect the reproductive rights of the immigrant population.

Significant differences were observed in the distribution of preterm births. Although immigrant women have a higher proportion of full-term births, they also have a higher proportion of extremely premature births. Haitians are the ones who exhibit the highest proportion of extreme prematurity. International studies have shown differences in the duration of pregnancy and prematurity in immigrant women, which may be influenced by socioeconomic, health and access to prenatal care factors (10, 17).

Although immigration has been associated with a higher risk of having COVID-19, in this study no differences were found regarding the prevalence of COVID-19 during pregnancy, or in the severity of the disease, admission to Intensive Care Unit, pneumonia, use of mechanical ventilation, prognosis, pulmonary thromboembolism, and hospitalization, between the immigrant and non-immigrant groups. However, these divergent results could be explained by the specific migratory conditions of the country since in Chile migration is a recent phenomenon and better perinatal maternal health indicators have been reported compared to the local population (12, 13). Furthermore, the importance of considering other contextual and sociocultural factors that could influence the observed outcomes is highlighted (7, 24–28).

The main limitations of this study includes a limited ability to extrapolate the results to other populations. Also, it should be noted that there might be a slight subestimation of the parameters, because as asymptomatic pregnant women were not tested within the first two months of the pandemic. Another limitation of the study is the used of secondary data, some relevant variables were not measured, such as maternal education, length of residence in the country, and the presence of pathologies that could influence the results. More studies should be conducted to address the effect of preeclampsia, and gestational diabetes and its impact on obstetric and perinatal outcomes.

## 5. Conclusion

These findings have significant implications for the perinatal care of the immigrant population, indicating that immigrant women may encounter additional barriers in terms of personalized care during childbirth, access to a companion, type of delivery, and establishing an emotional bond with their newborns. These differences could influence perinatal outcomes and underscore the need to address disparities in perinatal care between non-immigrant and immigrant pregnancy groups.

Furthermore, it is essential to consider other contextual and sociocultural factors that may be influencing the observed outcomes. These results should encourage additional investigation into the causes for these disparities in care and the development of targeted programs to enhance maternal health and obstetric care for immigrant women, not just in Chile but also in other nations, considering the specific stage of obstetric transition they are in.

## Data Availability

The original contributions presented in the study are included in the article/Supplementary Material, further inquiries can be directed to the corresponding author.
